# Everybody's Page

**Published:** 1891-01-24

**Authors:** 


### EVERYBODY'S PAGE.

The householder is only too familiar with the waste of gas which arises from the variability of pressure, and will hail with delight a regulator which checks the wasteful consumption at the burners when an excess of gas is delivered into the meter on the lights in shops being extinguished at night. By fitting up the regulator in question and connecting it with the tap on the meter supply pipe, the consumer can admit only such quantity of gas as is required.

The London Vegetarian Society seems to have met with sufficient success to enable it to widen its basis of operations. In conjunction with the Bread and Food Reform League an educational food fund and self-supporting halfpenny dinners have been established which aim at teaching the advantages of now much-neglected valuable foods. No doubt much benefit can be conferred on the poorer classes by bringing home to them the fact that a sustaining and wholesome diet exists which is within their means, though the claim on behalf of the committee that vegetarianism influences humanity on more points than any other subject of the day sounds somewhat extravagant.

Three well-known men have quite lately been cremated at Woking—Baron Huddleston, Mr. Kinglake, and the Duke of Bedford. Yearly the number of cremations increase, showing that the sanitary superiority of this method of disposing of the dead is becoming widely recognised. But besides the sanitary advantage, what ought to be impressed on the public is the moderate expense of cremation, no small consideration to those whose incomes are but moderate.

The incurable lepers of Venezuela appear to be in better case than are many who are victims to this dreadful disease. Though they are cut off from the mainland, and are all on one island about four miles from Maracaibo, their isolated life is made bearable for them. There is a building for them and one for the attendants, and besides this, those possessed of means are given land on which to build. There is a plentiful supply of water both for domestic and agricultural purposes, thus they are enabled to become tillers of the land. The island is stocked with game—artificially—there is a reading-room and a chapel, and occasional entertainments are provided.

